# Concurrent Serotonin Syndrome and Prolong QT Interval Induced by Paroxetine Overdose With Isotretinoin

**DOI:** 10.7759/cureus.14497

**Published:** 2021-04-15

**Authors:** Omar Al-Radideh, Kok Hoe Chan, Shawn Gupta, Jihad Slim, Sharath Bellary

**Affiliations:** 1 Internal Medicine, Saint Michael's Medical Center, Newark, USA; 2 Infectious Diseases, Saint Michael's Medical Center, Newark, USA; 3 Pulmonary/Critical Care, Saint Michael's Medical Center, Newark, USA

**Keywords:** paroxetine, serotonin syndrome, qt interval prolongation, isotretinoin, overdose

## Abstract

The use of serotonergic drugs has increased in the last decade especially selective serotonin reuptake inhibitors (SSRIs) with increased indications. Serotonin syndrome (SS) and QT prolongation are serious adverse reactions of SSRI use, they usually occur with concomitant use of two or more serotonergic medication. Herein, we are presenting an interesting unique case of SS and prolongation of QT interval after a suicidal attempt in a patient on isotretinoin with paroxetine overdosing. The prolongation of QT interval observed in this case could be related to isotretinoin synergistic effect. The risk of suicide and side effects of SSRI with isotretinoin, especially in patient with psychiatric illness would be a huge concern. This case hopes to raise the awareness of the risks when prescribing SSRI and isotretinoin in this group of patients.

## Introduction

Selective serotonin reuptake inhibitors (SSRIs) are commonly and widely used medications in clinical practice. Serotonin syndrome (SS) is one of the potentially lethal side effects of those medications [1]. The actual incidence and prevalence of SS is not well documented in the literature, likely due to low awareness of the symptomatology of SS, thus attributing the symptoms to other causes [2]. QT prolongation secondary to SSRIs have been well described in the literature. Nonetheless, among all SSRIs; paroxetine has the lowest risk of causing QT interval prolongation [3]. Treatment with isotretinoin has been described to exacerbate psychiatric disorders and placing the patient at a higher risk of suicide attempt [4]. Recognition and early management of this syndrome is paramount to prevent the life-threatening consequences. Herein, we are reporting a case of paroxetine overdose likely potentiated by isotretinoin which leads to SS in concurrent of seizure and cardiac toxicity with QT interval prolongation. 

## Case presentation

**Figure 1:**
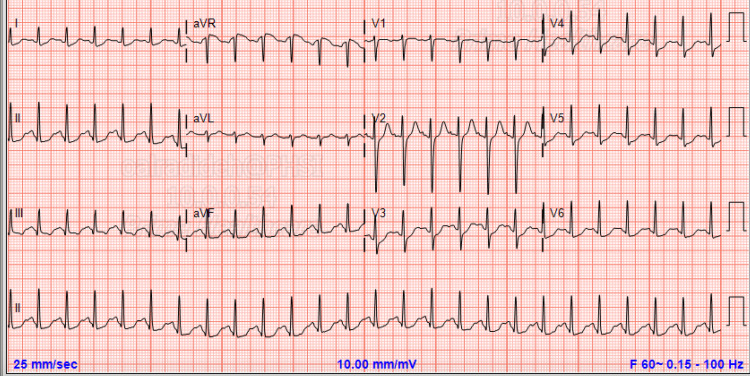
Initial ECG showed normal QT interval

A 20-year-old female with past medical history of post-traumatic stress syndrome on paroxetine 40 mg every day and facial acne on isotretinoin presented to the emergency department after two hours of intentional ingestion of seven pills of paroxetine in order to get the attention of her father after an argument. She denied the use of any recreational drug or ingestion of other medication. At the time of initial evaluation in the emergency department, she denied fever, vomiting, nausea, dizziness, seizure, headache, palpitation. States Toxicology Department was notified and in light of minimal symptoms, stable vital signs with no evidence of arrythmia on initial electrocardiogram (ECG) (Figure 1), decision was made for observation.

Six hours into observation, she complained of flushing and became restless and agitated. She sustained witnessed spontaneous aborted tonic clinic seizure activity of 90 seconds without tongue biting or urinary incontinence but with post ictal confusion. Her vital signs were notable for tachycardia with heart rate 130 beats/minute, tachypneic with respiratory rate 21 breaths/minute, blood pressure 123/88 mmHg, body temperature (37.6°C), and saturating 98% on room air. Physical exam evidenced agitation, restlessness, tremors noted mainly in lower extremities, hypertonia and hyperreflexia in lower extremities more noted in distal part and inducible clonus noted on bilateral feet. Other neurological exam was normal which included grossly normal cranial nerve, power of 5/5 in all extremities with intact sensation and negative posterior spinal Colum examination.

**Figure 2:**
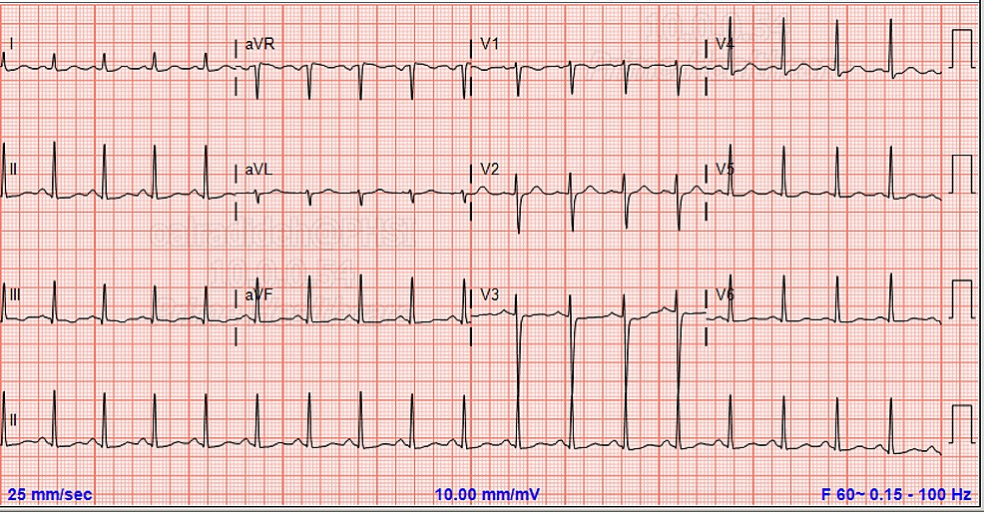
Repeat ECG showed prolonged corrected QT interval of >540 with normal PR interval and QRS complexes

Blood workup including the complete blood count and complete metabolic panel, troponin thyroid function test were within normal limits. CT of the head without contrast showed no abnormalities. ECG showed prolonged corrected QT interval of >540 with normal PR interval and QRS complexes (Figure 2).

Urine drug screen was negative and ethanol level was normal. After ruling out cerebral vascular insult and electrolytes abnormality, SS was suspected. Intravenous fluid and a dose of lorazepam 1 mg were given. States toxicology department was notified again and advised to start cyproheptadine 12 mg orally as single dose with 4 mg every eight hours as needed. She was admitted to intensive care unit for close monitoring with serial ECG. QT interval normalization was observed 16 hours later. No visualized seizures were noted during the hospitalization and other symptoms including hypertonia, hyperreflexia, agitation and inducible myoclonus were absent on repeat neurological exam. 

## Discussion

Paroxetine is an SSRI that has been approved in treatment of varieties of psychiatric illnesses including but not limited to depression, post-traumatic stress syndrome, eating disorders, generalized anxiety disorders and social phobia. The most common side effect of paroxetine includes dry mouth, sweating, sleep disturbance and sexual dysfunction [5].

Isotretinoin with its anti-inflammatory and immunomodulation characteristics has been approved for the treatment of comedonal acne [6]. The adverse side effects of isotretinoin include but are not limited to rash, teratogenicity and dry mouth [7,8]. The parallel relationship between isotretinoin and its risk of increasing suicide attempts has been a concern but the true causality is poorly described in the literature [4,9]. 

SS can happen in all age groups and there is no gender predilection. Over the years, the incidence of SS has been trending up likely related to more prescription of SSRI in clinical practice [10]. SS considered as a spectrum of manifestation that ranges from benign to life-threatening [11]. The most widely used clinical criteria for diagnosis of SS is the Hunter Toxicity Criteria Decision Rules with 84% sensitivity and 97% specificity. To fulfill the criteria patient should be on serotonergic agent and exhibits one of the following: (i) spontaneous clonus, (ii) tremor plus hyperreflexia, (iii) inducible clonus plus agitation, (iv) hypertonia plus temperature above 38ºC and ocular clonus, and (v) ocular clonus plus agitation [12]. 

The mechanisms behind SS mainly depend on alternation of serotonin metabolisms in the body which includes inhibition of re-uptake, increase synthesis, enhance and increase release and activation of serotonergic receptors [2]. Overdosing of a single SSRI rarely causes clinically significant symptoms [13]. Most of the fatalities involving SSRI overdose are related to massive ingestion of a single SSRI, or co-administration with another serotonergic agent [14,15]. 

Early recognition and promptly discontinuing the offending agent is the mainstay of treatment of SS. Patients are generally managed with supportive care with intravenous fluids and sedative medication with benzodiazepines for milder form of the disease [16]. In moderately ill patient, administration of serotonergic antagonist cyproheptadine which acts as histamine-1 receptor antagonist should be considered, this recommendation is based on a few case reports showing its success in alleviating the SS symptoms [17]. In patients with severe disease and hyperthermia (temperature >41 degree Celsius), tracheal intubation and mechanical ventilation along with sedation and paralytic agent is indicated [16]. 

Prolonged QT interval is defined as more than 470 milliseconds in post-pubertal male and 480 milliseconds in post-pubertal female [18]. Prolonged QT interval can lead to polymorphic ventricular tachycardia which is one of the life-threatening cardiac arrhythmia that leads to sudden death [19]. SSRI and its association with prolongation of QT interval are considered as dose-dependent mostly documented with Citalopram and Escitalopram as opposed to paroxetine [20]. We believe that our case represented a unique presentation of the most serious side effects of paroxetine with modesty dose ingestion of 280 mg (only represented seven times than the therapeutic doses recommended 40 mg/day) including SS with QT interval prolongation and tonic colonic seizure. Also, the concurrent use of isotretinoin and its possible association to increasing suicidal ideation might have played a role in her paroxetine overdose. 

## Conclusions

SSRI use considers safe with minimal side effect but overdosing of these medications could be life-threatening due to the risk of development of serotonin syndrome and cardiac toxicity. Patients who use isotretinoin and have a history of psychiatric illness must be closely follow-up and educated about the possibility of increasing suicide attempts.

## References

[1] Bartlett D (2017). Drug-induced serotonin syndrome. Crit Care Nurse.

[2] Boyer EW, Shannon M (2005). The serotonin syndrome. N Engl J Med.

[3] Funk KA, Bostwick JR (2013). A comparison of the risk of QT prolongation among SSRIs. Ann Pharmacother.

[4] Ludot M, Mouchabac S, Ferreri F (2015). Inter-relationships between isotretinoin treatment and psychiatric disorders: Depression, bipolar disorder, anxiety, psychosis and suicide risks. World J Psychiatry.

[5] Shrestha P, Fariba K, Abdijadid S (2021). Paroxetine. https://www.ncbi.nlm.nih.gov/books/NBK526022/.

[6] Abdelmaksoud A, Lotti T, Anadolu R (2020). Low dose of isotretinoin: a comprehensive review. Dermatol Ther.

[7] Blasiak RC, Stamey CR, Burkhart CN, Lugo-Somolinos A, Morrell DS (2013). High-dose isotretinoin treatment and the rate of retrial, relapse, and adverse effects in patients with acne vulgaris. JAMA Dermatol.

[8] Vallerand IA, Lewinson RT, Farris MS, Sibley CD, Ramien ML, Bulloch AGM, Patten SB (2018). Efficacy and adverse events of oral isotretinoin for acne: a systematic review. Br J Dermatol.

[9] Sundström A, Alfredsson L, Sjölin-Forsberg G, Gerdén B, Bergman U, Jokinen J (2010). Association of suicide attempts with acne and treatment with isotretinoin: retrospective Swedish cohort study. BMJ.

[10] Mason PJ, Morris VA, Balcezak TJ (2000). Serotonin syndrome. Presentation of 2 cases and review of the literature. Medicine.

[11] Birmes P, Coppin D, Schmitt L, Lauque D (2003). Serotonin syndrome: a brief review. CMAJ.

[12] Dunkley EJ, Isbister GK, Sibbritt D, Dawson AH, Whyte IM (2003). The Hunter Serotonin Toxicity Criteria: simple and accurate diagnostic decision rules for serotonin toxicity. QJM.

[13] Barbey JT, Roose SP (1998). SSRI safety in overdose. J Clin Psychiatry.

[14] Muzyk AJ, Jakel RJ, Preud’homme X (2010). Serotonin syndrome after a massive overdose of controlled-release paroxetine. Psychosomatics.

[15] Peacock LE, Wright F (2011). Serotonin syndrome secondary to tramadol and citalopram. Age Ageing.

[16] Wang RZ, Vashistha V, Kaur S, Houchens NW (2016). Serotonin syndrome: preventing, recognizing, and treating it. Cleve Clin J Med.

[17] Kapur S, Zipursky RB, Jones C, Wilson AA, DaSilva JD, Houle S (1997). Cyproheptadine: a potent in vivo serotonin antagonist. Am J Psychiatry.

[18] Drew BJ, Ackerman MJ, Funk M (2010). Prevention of torsade de pointes in hospital settings: a scientific statement from the American Heart Association and the American College of Cardiology Foundation. Circulation.

[19] Haddad PM, Anderson IM (2002). Antipsychotic-related QTc prolongation, torsade de pointes and sudden death. Drugs.

[20] Beach SR, Kostis WJ, Celano CM, Januzzi JL, Ruskin JN, Noseworthy PA, Huffman JC (2014). Meta-analysis of selective serotonin reuptake inhibitor-associated QTc prolongation. J Clin Psychiatry.

